# Kawasaki disease among Egyptian children: A case series

**DOI:** 10.21542/gcsp.2017.25

**Published:** 2017-10-31

**Authors:** Tarek Hamed Attia, Saed M. Morsy, Bashier A. Hassan, Al Shymaa A. Ali

**Affiliations:** Pediatrics Department, Faculty of Medicine, Zagazig University, Zagazig, Egypt

## Abstract

Kawasaki disease is an acute vasculitis of early childhood. Its incidence varies among different ethnic groups with higher rates among Asians. In this case series, we presented four cases of Kawasaki disease with incomplete or atypical presentations in Egyptian children.

Two cases presented with meningitis, which is not a criteria for the diagnosis of Kawasaki disease. The other two cases presented with pharyngitis and fever, which did not respond to antibiotics. The clinical criteria for diagnosis of Kawasaki disease were either incomplete or appeared sequentially. Coronary artery aneurysms were detected in one case, while the others had normal coronary by echocardiography. All cases were followed in our clinic, according to international guidelines.

Early diagnosis and management of Kawasaki disease are important to ensure a good outcome and a high index of suspicion in febrile children is required irrespective of the clinical presentation.

## Introduction

In most countries, Kawasaki disease (KD) has replaced acute rheumatic fever as the leading cause of childhood-acquired heart disease^1–3^. It occurs most often in babies and children, aged 6 months to 5 years^4^.

The American Heart Association (AHA) had developed guidelines for diagnosing complete as well as incomplete Kawasaki. Typical KD is diagnosed if patients have fever persisting at least 5 days, plus the presence of at least 4 of the following 5 features: bilateral non-suppurative conjunctival injection; changes in the lips and oral cavity (cracked and erythematous lips, strawberry tongue and injection of oropharyngeal mucosa); cervical lymphadenopathy, extremity changes (erythema and edema of hands and feet followed by desquamation of fingertips), and polymorphous rash^5^.

Some patients who do not fulfill the classic criteria for KD have been diagnosed as having “incomplete” or atypical KD^5^. In certain situations, KD can be a very challenging diagnosis^3^ or even be missed.

Documented data about KD among Egyptian children is very scarce. We hereby present four cases of KD with incomplete or atypical presentations in Egyptian children.

## Case series

### Case 1

An 11-year-old male presented with high fever for 3 days with nausea and vomiting. Upon admission, he had convulsions with neck stiffness, positive Brudzinski’s and Kernig’s signs, a clinical picture suggesting a case of meningitis. Examinations of other systems were unremarkable. Treatment with antibiotics for presumed meningitis was started, but fever did not respond and it was not until the sixth day of fever when bilateral non-purulent conjunctival injection as well as a maculopapular skin rash on the face, trunk and extremities appeared.

Analysis of cerebrospinal fluid (CSF) showed a white blood cell count (WBC) of 4/mm^3^. Levels of CSF glucose and protein were within normal ranges. Cerebrospinal fluid viral screen was negative. Complete blood count (CBC) showed leucocytosis and neutrophilia with normal platelet count. C-reactive protein (CRP) was 24 mg/L and first hour erythrocyte sedimentation rate (ESR) was 48 mm/h. Liver function tests revealed elevated liver enzymes and low albumin level. Other laboratory investigation revealed negative CSF, urine, throat and blood culture, negative metabolic screening, and normal urine analysis. In addition, echocardiographic examination was normal.

**Figure 1.**
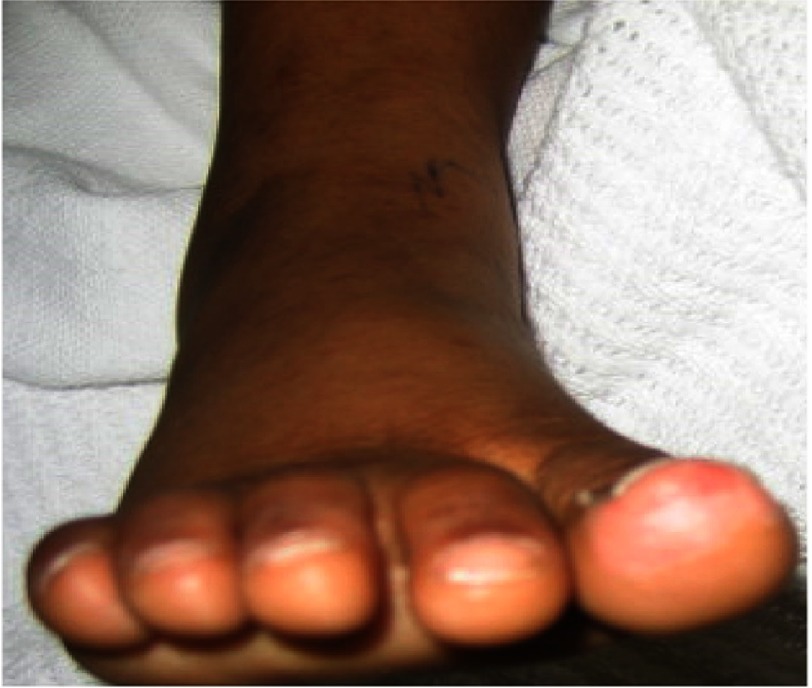
Case 1 with fingertips desquamation.

Based on clinical findings, laboratory results, and exclusion of possible other etiologies, KD was suspected with a rare presentation of meningitis. Echocardiography was normal. Intravenous immunoglobulin (IVIG) 2 g/kg and aspirin 100 mg/kg per day orally were started. Body temperature was normalized 30 hours after completion of IVIG infusion. On the 7th day of fever, there was thrombocytosis and on day 11 the skin started to desquamate and peeling appeared at the fingers tips and perineal region (Figure 1). High dose aspirin was continued until the 14th day of illness. After that, low-dose aspirin at 5 mg/kg per day was given as a single dose for 4 weeks; during which time acute phase reactant, CBC and serum transaminases were gradually normalized.

Repeated echocardiographic examinations revealed no cardiac abnormalities and repeated follow up visits, over the following 3 years, revealed a healthy child.

### Case 2

A 3.5-month-old male infant presented with fever and convulsion. On examination, he was drowsy and irritable. The anterior fontanel was bulging and pulsating. The patient was diagnosed provisionally as a case of meningitis. Triple therapy was started (ceftriaxone, vancomycin and acyclovir) and dexamethasone was planned for 2 days awaiting culture results. CSF examination was in favor of aseptic meningitis; WBC was 2/mm^3^, CSF glucose was 60 mg/dl while blood glucose was 94 mg/dl and CSF protein was 20 mg/dl.

Viral screening from CSF was negative for herpes virus and enterovirus. Results of blood, CSF, throat and urine bacterial cultures and serological tests for bacteria and viruses were negative. Metabolic screening was negative. A daily clinical evaluation was done. On day four of fever, non-purulent conjunctivitis was noted. On the next day, macular rash appeared on truck, chest and face. Fever did not resolve until the 6th day. With KD in mind, liver function, repeated CBC and urine analysis were performed. There was leukocytosis, thrombocytosis, anemia for age and low albumin level. CRP was 20 mg/L and ESR was 40 mm/h one hour after admission and repeated on day 6 (CRP 30 mg/l and ESR 60 mm/h). Atypical Kawasaki was diagnosed in spite of normal echocardiography. IVIG and aspirin were started and antibiotics were discontinued with subsidence of fever within 30 hours of starting IVIG. The child was followed regularly in the cardiology clinic for 2 years with no signs of coronary artery involvement.

### Case 3

A 5-year-old boy was diagnosed with pharyngitis and received oral antibiotics. The fever persisted and he was evaluated for viral etiology of pharyngitis as well as other causes of fever. Viral screening was negative and other laboratory tests were insignificant.

He was referred to our hospital for further evaluation on the 10^th^ day of fever. Fever was 39°C, with bilateral non purulent conjunctivitis, red lips with congested throat and there was erythema of the hands and feet.

Upon admission, laboratory studies revealed a white blood cell count of 18,500/mm^3^ with 72% neutrophils. anemia for age (hemoglobin 10 g/dL) and thrombocytosis (platelet count of 850,000/mm^3^). CRP was 58 mg/L and first hour ESR was 84 mm/h. Serum aspartate transaminase (458 U/L), alanine transferase (385 U/L) were high, while serum albumin was low. Urine analysis was normal. Results of blood, CSF, throat and urine bacterial cultures and serological tests for bacteria and viruses were negative.

**Figure 2.**
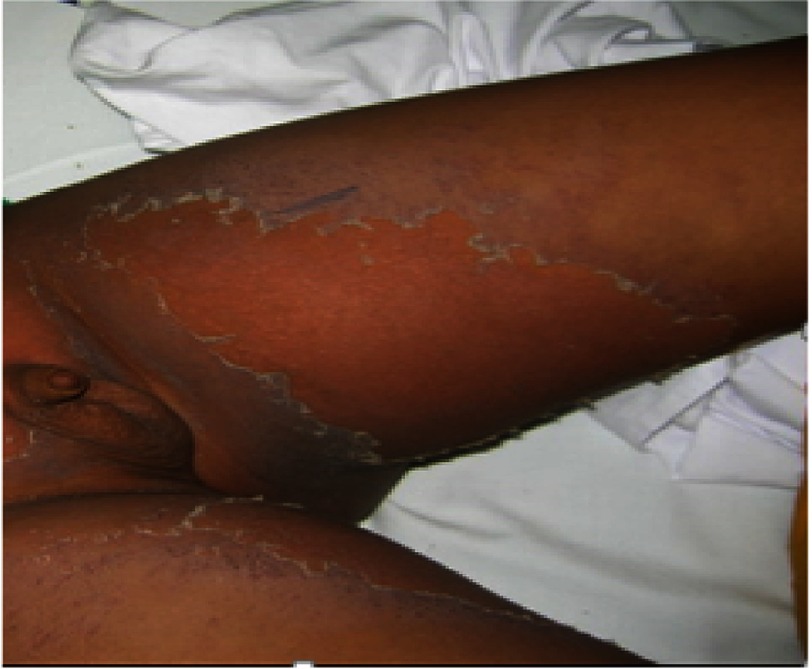
Case 3 with periungual desquamation.

**Figure 3.**
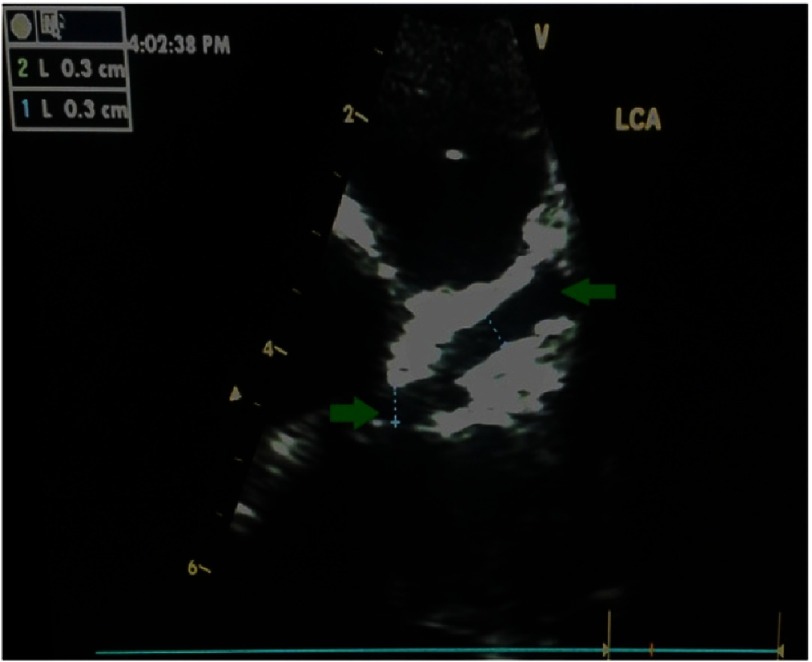
Two-dimensional echocardiography of case 3 showing saccular dilatation of the origin of left main coronary artery (LCA) (arrow) and aneurysmal dilatation of left anterior descending (LAD) artery.

A diagnosis of atypical Kawasaki was considered and echocardiography was requested. The day after admission, he had desquamation of the skin at his fingertips and perianal region (Figure 2). Echocardiographic examination revealed saccular dilatation of the origin of left main coronary artery (LCA) and aneurysmal dilatation of left anterior descending (LAD) artery (Figure 3).

Intravenous immunoglobulin (2 g/kg) for 12 hours and high-dosage (100 mg/kg per day) acetylsalicylic acid treatment were administered. Fever subsided after 28 hours of IVIG therapy, and acute phase reactants, including ESR and CRP, were normalized within 25 days.

The patient was discharged on low-dosage acetylsalicylic acid (5 mg/kg per day). With monthly follow-up echocardiography. After 6 months, echocardiographic examination revealed normal coronaries with no aneurysmal dilatation.

### Case 4

**Figure 4.**
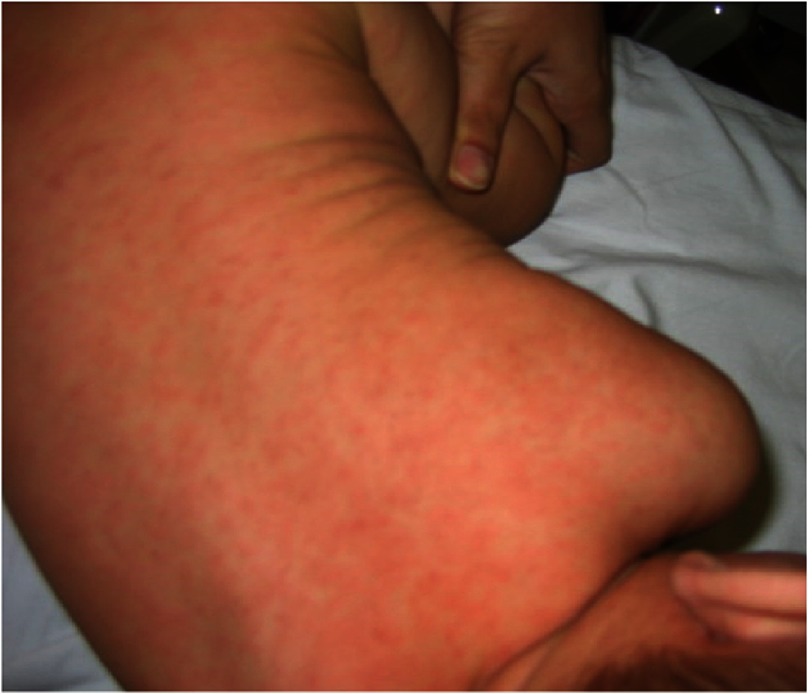
Case 4 with diffuse erythematous eruption on trunk.

A 1-year-old boy presented with fever for 7 days. He was treated for pharyngitis with antibiotics. He developed a nonspecific skin rash diagnosed as a drug eruption for which the antibiotic was changed to azithromycin with an added antihistamine (Figure 4). Upon admission, the examination revealed congested throat, hyperemic tongue, stomatitis and bilateral enlarged cervical lymph nodes. Nothing of note was found in the rest of examination.

Investigations were requested, including CBC, CRP, and ESR, urine analysis, cultures and viral screening. One next day the child developed non-suppurative conjunctival hyperemia and macular rash on the face and trunk. One day later, erythema of hands and feet and peeling of the fingers started.

CBC revealed anemia (hemoglobin 9.8 g/dL), neutrophilic leukocytosis (white blood cells 16,200/mm^3^, neutrophils 78%), and an elevated platelet count of 720/mm^3^. C-reactive protein was (44 mg/L) and erythrocyte sedimentation rate (68 mm/hr.). Serum aspartate transaminase (670 U/L), alanine transferase (712 U/L) were high, while serum albumin was normal. Urine analysis was normal. Results of blood, throat and urine bacterial cultures and serological tests for bacteria and viruses were negative.

A diagnosis of typical Kawasaki was considered. Echocardiographic examination was normal. Intravenous immunoglobulin (2 g/kg) over 12 hours and aspirin (100 mg/kg per day) were started. Fever disappeared one day after IVIG and ESR and CRP were normalized within 4 weeks. The patient was followed up for one year with normal echocardiograms.

## Discussion

Kawasaki disease is a difficult clinical diagnosis^5^. Firstly, there is an absence of diagnostic tests and we must rely on clinical criteria for diagnosis. Secondly, clinical criteria may manifest sequentially, rather than simultaneously, which may delay diagnosis . Thirdly, there is variability in patient presentation, as many symptoms may accompany principal criteria such as arthritis, gastrointestinal symptoms, extreme irritability, hydrops and anterior uveitis. Fourthly, patients who present with incomplete KD never develop all the classic criteria. Finally, there is a misconception amongst many Egyptian clinicians that KD is uncommon in Egypt. All these factors may contribute to missing the diagnosis or treatment delay^6,7^.

The current case series presents some difficulties that may hamper an accurate*,* timely diagnosis of KD. In cases 1 and 2, the initial presentation was aseptic meningitis, which is a rare presentation for KD. Also, principal features of classical KD were not fulfilled. Several case reports document this rare presentation^8–10^. Pathogenesis of aseptic meningitis in KD is unclear. In case 3, the diagnosis was missed for 10 days and the delay in diagnosis and treatment resulted in aneurysms of the coronary arteries. It was reported that the features of KD may present sequentially rather than simultaneously which leads to more delay in diagnosis as in this case^6^. Case 4 represents another one of the dilemmas faced by clinicians during KD diagnosis even when presentation is classical. A high index of suspicion is needed to diagnose and treat KD early during the acute phase in order to decrease the risk of coronary artery abnormalities.
